# Medical and Philosophical News

**Published:** 1784

**Authors:** 


### SECTION III.

### Medical and Philosophical News.

THE Royal Academy of Sciences at Paris, not having received any ſatisfactory anſwer to their inquiries concerning the decompoſition of ſea ſalt (ſee our 3d vol. p. 86) have propoſed it a ſecond time. Diſſertations on this ſubject will be received till Eaſter 1785.

The Academy have been equally unſucceſsful on the ſubject of the lymphatic ſyſtem (ſee vol. 3, p. 306). In its ſtead, therefore, they now require a deſcription of the intercoſtal nerve in man. Many parts of this nerve are already well known; but a great number of its ramifications cations ſtill remain to be deſcribed with accuracy. The Academy give notice, that care will be taken to aſcertain the truth of all the new obſervations they ſhall receive on the ſubject, ſo that no imaginary deſcriptions will be admitted. Drawings are not abſolutely required; but, if accurate, will add to the value of the diſſertations in the eſtimation of the Academy.

The Academy, thinking it may be uſeful to propoſe two ſubjects which may ſerve to illuſtrate each other, offer another premium for the beſt deſcription of the intercoſtal nerve in animals, particularly in the monkey, dog, ſheep, turkey, frog, and carp, as affording the greateſt variety of ſtructure. On deſcriptions taken from the monkey, however, they do not abſolutely inſiſt. Each of theſe premiums is to be of 1500 livres value; the diſſertations muſt be written either in Latin or French, and ſent to the ſecretary before the 11th of Nov. 1785.

---

At a public meeting of the Royal Medical Society at Paris, on the 2d of March, 1784, the premium of 400 livres offered by the Society for the beſt diſſertation on the diſeaſes of an army during the ſummer, and in very hot climates, mates (fee vol. 3, p. 85) was adjudged to Dr. Thion de la Chaume, late phyfician to the army in Minorca.—And the premium of 600 livres for the beft treatife on the different fpecies and cafes of dropfy, in which the ufe of diluents or a dry regimen ought to be preferred (fee vol. 3, 419) was divided between different candidates in proportion to the fuppofed merits of their refpective differtations; viz. to Dr. Mezler, phyfician to the reigning count of Lipinghen-Nippenbourg, 300 livres; and to Dr. Chartier, phyfician to his R. H. the count de Provence, Dr. Thomas Olliff, an Englifh phyfician, now at Paris, and Dr. Peter Matthew Nielen, of Utrecht, 100 livres each.

The Society now announce the following fubject for a prize-medal, viz. "Of the four annual conftitutions admitted by the ancients, "and which are the catarrhous, the inflammatory, "the bilious, and the atrabilious, the three firft "being known and well afcertained, the quef- "tion is, whether the fourth hath a diftinct "exiftence, and what fhare it has in the pro- "duction of epidemical difeafes?" Differtations on this fubject muft be fent to M. Vicq d'Azyr, fecretary to the fociety, before May 1, 1785.

At the above meeting were read the eulogies of Dr. Hunter and Dr. Sanchés, two foreign aſſociates of the ſociety, lately deceaſed; and likewiſe a paper by M. Thouret, on the ſtructure of the poſterior ſymphyſes of the pelvis, and on the mechaniſm of their ſeparation in parturition.

---

The following queſtion is propoſed by the Royal Academy of Sciences at Dijon, for a prize of 300 livres, viz. " What are the ſymp-" toms which at the beginning of a continued " or an intermittent fever, will enable the phy-" ſician to judge whether ſuch fever will be ma-" lignant; and what are the ſigns which in the " courſe of the fever will indicate the preciſe " time when it is on the point of aſſuming a " malignant character?"—The diſſertations on this ſubject muſt be ſent to M. Maret, ſecretary to the academy, before the 1ſt of April 1785.

---

The Dutch Society of Sciences at Harlem offer a premium of a gold medal to the perſon who, by very accurate and ſatisfactory experiments, ſhall determine " How far Dr. Crawford's

" ford's theory of fire and heat deserves to be " admitted or rejected; and if the truth of that " theory, either wholly or in part, is established " by experiments, how far it advances us in " the philosophy of fire?" —Dissertations on this subject will be received by the secretary at Harlem till January 1, 1786.

---

Mr. Cavendish, in a very ingenious paper read lately at the Royal Society, has related experiments to prove that the diminution of common air by phlogistication is not owing to the generation or separation of fixed air from it, as hath been commonly supposed, but to the production of water formed by the combination of the phlogiston, disengaged in the process, with the dephlogisticated part of common air. On firing a mixture of inflammable and dephlogisticated airs in close vessels, in the manner first suggested by Mr. Warltire (see vol. II. p. 102.), Mr. Cavendish has found, that the two airs, thus mixed and exploded, are almost wholly converted into water. The experiments of this very respectable philosopher have convinced him, that dephlogisticated and phlogisticated airs are not to be considered as the same fluid, differing only in degree of

 phlophlogistication, but as two distinct substances, the union of which forms common atmospheric air. He contends, that phlogisticated air is nothing more than nitrous acid combined with phlogiston; and he is inclined to think, that inflammable air is not pure phlogiston, as Dr. Priestley and after him Mr. Kirwan have asserted, but rather water united to phlogiston. Since this Essay, of which we pretend here to give only the principal outlines from memory, was read, Mr. Kirwan, in a paper of considerable length, read also at the Royal Society, has, with his usual ability, defended his opinion concerning the generation of fixed air in phlogisticating processes, and likewise concerning the identity of inflammable air and phlogiston. These observations have been followed by a reply from Mr. Cavendish.

---

The perfection of telescopes, and other optical instruments, has hitherto been impeded by the different refrangibility of the rays of light, the prismatic colours occasioned thereby, rendering the images of objects more or less confused. It is well known, however, that those rays which fall perpendicularly on a surface, are transmitted without any refraction. If these rays, rays, therefore, can be feparated from thofe which fall obliquely, dioptrical inftruments may be brought to as great perfection with regard to the diftinctnefs of the images, as catroptrical ones, and in point of luminoufnefs much more fo. This has at length been effected by an ingenious artift in London, who has already conftructed a great variety of inftruments on this principle.

---

A cafe of hydrophobia has lately occurred in Weftminfter, which may ferve as an additional proof of the inefficacy of the Ormfkirk remedy. The patient (Mr.Dawfan, grocer, inBarton-ftreet) having been bit in one his thumbs by a favourite dog that was fuppofed to be mad, began the ufe of this medicine the next day, and perfevered in it in the ufual manner. The wound in his thumb healed, and he felt no inconvenience from it till Monday the 8th of March, the forty-fecond day after the bite, when he began to complain of a tingling in the part that had been bit, and faid his arm felt ftiff and uneafy, which he attributed to rheumatifm. The fymptoms of hydrophobia came on the next day about noon, and he died on Thurfday, the 11th, in the afternoon.

---

The

The Abbé Mann (a native of this country and a very reſpectable member of the Academy of Sciences at Bruſſels, where he now reſides) of whom ſome mention was made in a former volume of our Journal (vol. II. p. 410) as having been cured of the gout by the uſe of the extract of cicuta, has publiſhed a very full account of his caſe in the *Eſprit des Journaux* for February 1784, from which we have collected the following particulars for the information of our readers. The learned Abbé, it ſeems, paſſed ſeveral years of the early part of his life in the Spaniſh ſervice; but a love of retirement and ſtudy induced him, at the age of five-and-twenty, to reſign his military employment, and enter into a convent of Carthuſians, at Nieuport in Flanders, of which he afterwards became Superior. In 1763, being then in his 29th year, he began to be attacked with the gout. The change of climate he had experienced by removing from Spain to Flanders; the exceſſive cold he was expoſed to in winter, by paſſing conſtantly ſeven or eight hours of the day in the church; his cloſe application to ſtudy, and his want of exerciſe, all contributed to increaſe his diſpoſition to this diſeaſe, which returned at intervals, and at length became ſo frequent, that from 1768 till

1779 he did not paſs a year without having three or four ſevere fits.

In the ſummer of 1772 the peruſal of Dr. Cadogan's pamphlet on the gout induced him to adopt the regimen recommended by that writer, and he adhered to it ſtrictly for the ſpace of four months. Twice during that time the gout appeared ſlightly, but continued only a day. He now found himſelf in a very infirm ſtate, and at length, in the month of September, the gout attacked his ſtomach, breaſt, and head. This, which was by far the moſt violent and dangerous paroxyſm he had ever experienced, laſted ſeven months.

In 1778 he quitted the order of Carthuſians, became a ſecular prieſt; and removed to Bruſſels, but without experiencing any conſiderable amendment in his health. He now became ſubject to violent ſpaſms, but he acknowledges that his gouty paroxyſms were leſs violent than that of 1772.

Such was the ſtate of his caſe in the ſpring of 1779, when he was adviſed by Mr. Himelbaur, ſurgeon at Bruſſels, to try the effect of the extracts of cicuta and aconitum, procured from Vienna, and taken in pills of two grains each. At firſt he took the cicuta alone three times a day, ſwallowing lowing four pills, or eight grains of the extract, each time. By degrees he increaſed the doſe and added a pill of aconitum. At length, after five or ſix months, he took daily, of theſe two extracts, 100 or 120 grains; but commonly in the proportion of one pill of aconitum to five or ſix of cicuta, and in this ſame proportion he ſtill continues to take them, though in much leſs quantity than during the two firſt years. To theſe two extracts he added the uſe of a camphorated julep, and from time to time he has taken a doſe of rhubarb. Theſe are the only remedies he has made uſe of during the laſt four years, and he has obſerved no particular regimen, except that he has been careful to take only ſuch kind of food as is eaſy of digeſtion.

During the firſt three months of his taking the cicuta he experienced no ſenſible effect from it, either good or bad. His ſurgeon then adviſed him to add the aconitum, and ſoon after he began to experience a degree of eaſe in his joints he had long been a ſtranger to. His appetite improved, and his ſpaſms, and other ſymptoms of morbid irritability, diminiſhed very ſenſibly.

This encouraged him to perſevere in the uſe of his remedy, and before the winter came on he he took it in the largeſt doſes. During that and the winter of 1780-81 he was ſeveral times threatened with a return of the gout, but had no fit, and ſince the laſt-mentioned period he has been perfectly free from the diſeaſe. He has lately had an eryſipelatous inflammation on one of his legs, which was ſoon diſſipated, and he is now in perfect health, and uſes a great deal of exerciſe, being able to walk ten or twelve miles without fatigue.

---

Extract of a letter from Mr. Thomas Henry, F. R. S. to Dr. Simmons, dated Mancheſter, March 10, 1784.

" The preſent winter has afforded ample opportunities for repeating Dr. Black's experiments on latent heat. One of them which I accidentally made was attended with a circumſtance ſomewhat remarkable. Two four ounce vials were left during the Chriſtmas receſs in the ſame window, in my lecture-room, next the ſtreet, where great numbers of carriages are conſtantly paſſing. The one contained ſand and water; the other mercury ℥ij and water. The water upon the ſand was completely frozen; that over the mercury had no ſign of congelation;

 but but on my ſhaking the mercury and water violently together, the water inſtantly became an almoſt ſolid cake of ice.——Would one not have expected, that the action of the mercury would have impeded the chryſtalliſation of the water by diſturbing the arrangement of the particles?

"Mr. Wedgewood writes me word, that he has made trial of M. M. Lavoiſier and de la Place's method of meaſuring heat (deſcribed in the London Medical Journal, vol. IV. p. 235.) as an intermediate meaſure of heat for connecting his thermometer with Fahrenheit's, but could not make it ſucceed; for a part of the ice which is melted by the heated body is abſorbed and detained by the unmelted part; and, what ſeems extraordinary, a new congelation takes place even in the melting ice. He has alſo finiſhed a ſet of experiments for the ſame purpoſe on another principle, viz. the expanſion of metals, in which he met with ſeveral difficulties, but it proved more ſatisfactory, though perhaps not ſo exact as might be wiſhed. He intends to communicate both to the Royal Society."

---

Count Auguſtine Caſati, member of the Royal Academy of Sciences at Berlin, has communicated cated to the college of phyficians of that capital, a remedy which is faid to be of great efficacy in the rickets. It is the root of the *ofmunda regalis* or *flowering fern* given in decoction, powder, or extract.

---

We formerly mentioned the infpiffated juice of *faponaria officinalis* or *fopewort* as having been adminiftered with fuccefs in cafes of gonorrhœa. (See vol. I. p. 433.) In the *Journal General de France* (a new Journal lately fet on foot at Paris) for Feb. 14, 1784, M. Segey, one of the phyficians to the king of France, has publifhed the manner in which he has fucceeded with this plant in the moft obftinate cafes of *lues venerea.* He directs an ounce and a half of the dried root, and half an ounce of the dry plant, to be boiled in three quarts of water to two. One or two quarts of this decoction are to be taken daily, and in bad cafes, the patient is to take at the fame time the plant in powder, or in the form of an extract. The fame plant is likewife faid to have been ufeful when applied externally to venereal ulcers, either in fomentation, or in a dry form by fprinkling it in powder over the fores.

---

We are indebted to M. Andry, Doctor Regent of the Faculty of Phyfic at Paris, and to Mr. de Magellan, F. R. S. for the following anecdotes of the late Dr. Sanchés, whofe death we announced in our laft number (vol. 4, p. 432)—This learned phyfician was born on the 7th of March, 1699, at Penna-Macor, in Portugal. His father, who was an opulent merchant, and intended him for the bar, gave him a liberal education; but being difpleafed at finding him at the age of eighteen obftinately bent on the profeffion of phyfic, withdrew his protection, and he was indebted, it feems, to Dr. Nunés Ribeiro, his mother's brother, who was a phyfician of confiderable repute at Lifbon, for the means of profecuting his medical ftudies, which he did firft at Coimbra, and afterwards at Salamanca, where he took the degree of Doctor of Phyfic in 1724; and the year following he procured the appointment of phyfician to the town of Benevente in Portugal, for which, as is the cuftom of that country, he had a fmall penfion. His ftay at this place, however, was but fhort. He was defirous of feeing more of the world, and of improving himfelf in his profeffion. With this view he came and paffed two years in London, and had even even an intention of fixing here, but a bad ſtate of health, which he attributed to the climate, induced him to return to the continent. Soon after we find him proſecuting his medical ſtudies at Leyden under the celebrated Boerhaave, and it will be a ſufficient proof of his diligence and merit to obſerve, that in 1731, when the empreſs of Ruſſia requeſted Boerhaave to recommend to her three phyſicians, the profeſſor immediately fixed upon Dr. Sanchés to be one of the number.

Juſt as he was ſetting out for Ruſſia, he was informed that his father was lately dead, and that his mother, in an unſucceſsful law-ſuit with the Portugueſe admiralty, had loſt the greater part of her fortune. He immediately aſſigned over his own little claims and expectations in Portugal for her ſupport. Soon after his arrival at Peterſburgh Dr. Bidloo (ſon of the famous phyſician of that name) who was at that time firſt phyſician to the empreſs, gave him an appointment in the hoſpital at Moſcow, where he remained till 1734, when he was employed as phyſician to the army, in which capacity he was preſent at the ſiege of Aſoph, where he was attacked with a dangerous fever, and when he began to recover found himſelf in a tent, tent, abandoned by his attendants, and plundered of his papers and effects.

In 1740 he was appointed one of the phyſicians to the court, and conſulted by the empreſs, who had for eight years been labouring under a diſeaſe, the cauſe of which had never been ſatisfactorily aſcertained. Dr. Sanchés, in a converſation with the prime miniſter, gave it as his opinion that the complaint originated from a ſtone in one of the kidnies, and admitted only of palliation. At the end of ſix months the empreſs died, and the truth of his opinion was confirmed by diſſection.

Soon after the death of the empreſs, Dr. Sanchés was advanced by the regent to the office of firſt phyſician, but the revolution of 1742, which placed Elizabeth Petrowna on the throne, deprived him of all his appointments. Hardly a day paſſed that he did not hear of ſome of his friends periſhing on the ſcaffold, and it was not without much difficulty that he obtained leave to retire from Ruſſia. His library, which had coſt him 1200 pounds ſterling, he diſpoſed of to the academy of St. Peterſburgh, of which he was an honorary member, and in return they agreed to give him a penſion of forty pounds per annum.

During

During his refidence in Ruffia he had availed himfelf of his fituation at court, to eftablifh a correfpondence with the Jefuits in China, who, in return for books of aftronomy and other prefents, fent him feeds and plants, together with other articles of natural hiftory. It was from Dr. Sanchés that the late Mr. Peter Colinfon firft received the feeds of the true rhubarb, but the plants were deftroyed by fome accident, and it was not till feveral years afterwards that rhubarb was cultivated with fuccefs in this country from feeds fent over by the late Dr. Mounfey.

In 1747 he went to refide at Paris, where he remained till his death. He enjoyed the friendfhip of the moft celebrated phyficians and philofophers of that capital, and at the inftitution of a Royal Medical Society he was chofen a foreign affociate. He was likewife a member of the Royal Academy of Lifbon, to the eftablifhment of which his advice had probably contributed, as he drew up, at the defire of the court of Portugal, feveral memorials on the plans neceffary to be adopted for the encouragement of fcience. Some of thefe papers, relative to the eftablifhment of a univerfity, were printed during his life-time in Portuguefe, and the reft have been found among his manufcripts.

His

His ſervices in Ruſſia remained for ſixteen years unnoticed; but when the preſent empreſs aſcended the throne Dr. Sanchés was not forgotten. He had attended her in a dangerous illneſs when ſhe was very young, and ſhe now rewarded him with a penſion of a thouſand róubles, which was punctually paid till his death. He likewiſe received a penſion from the court of Portugal, and another from prince Gallitzin. A great part of this income he employed in acts of benevolence. Of the liberality with which he adminiſtered to the wants of his relations and friends, ſeveral ſtriking inſtances, which the limits of our Journal will not permit us to inſert, have been related to us by Mr. de Magellan.

He was naturally of an infirm habit of body, and, during the laſt thirty years of his life, frequently voided ſmall ſtones with his urine. The diſpoſition to this diſeaſe increaſed as he advanced in years, and for a conſiderable time before his death he was confined to his apartments. The laſt viſit he made was, in 1782, to the grand duke of Ruſſia, who was then at Paris. In September 1783, he perceived that his end was approaching, and he died on the 14th of October following. His library, which was conconfiderable, he has bequeathed to his brother, Dr. Marcello Sanchés, who was likewife a pupil of Boerhaave, and who refides at Naples. His manufcripts (among which, befides a confiderable number of papers on medical fubjects, are letters written to him by Boerhaave, Van Swieten, Gaubius, Haller, Werlhof, Pringle, Fothergill, and other learned men) are in the poffeffion of Dr. Andry. His printed works, on the origin of the venereal difeafe and other fubjects, are well known to medical readers; but his knowledge, it feems, was not confined to his own profeffion; he poffeffed a fund of general learning, and is faid to have been profoundly verfed in politics.

---

It is in compliance with the requeft of fome of our correfpondents, rather than from any favourable opinion we ourfelves have of the remedy, that we here give fome account of a fpecific lately publifhed in Spain for the cure of cancers (fee vol. 4, p. 323). This remedy, which is faid to have been long in ufe amongft the Indians of Guatimala, in South America, confifts in fwallowing one, two, or three raw lizards every morning fafting, after fkinning them and cutting off their heads and tails. The little lizard known by the name of *Lacerta vulgaris*, and which the French call *Anoli de terre*, or *Gobe mouche*, is the ſpecies recommended for this purpoſe. This remedy is ſaid to have produced good effects, not only in cancers, but likewiſe in leproſy, and the venereal diſeaſe.

---

*New works about to be publiſhed.*——1. A Treatiſe on Fevers, by Dr. John Gardiner, Fellow of the Royal College of Phyſicians at Edinburgh. —2. A work on poiſons, by Dr. Houlſton, phyſician at Liverpool.

